# AI algorithm for personalized resource allocation and treatment of hemorrhage casualties

**DOI:** 10.3389/fphys.2024.1327948

**Published:** 2024-01-25

**Authors:** Xin Jin, Andrew Frock, Sridevi Nagaraja, Anders Wallqvist, Jaques Reifman

**Affiliations:** ^1^ Department of Defense Biotechnology High Performance Computing Software Applications Institute, Telemedicine and Advanced Technology Research Center, United States Army Medical Research and Development Command, Fort Detrick, MD, United States; ^2^ The Henry M. Jackson Foundation for the Advancement of Military Medicine Inc., Bethesda, MD, United States

**Keywords:** artificial intelligence, fluid resuscitation, hemorrhage, resource utilization, trauma

## Abstract

A deep neural network-based artificial intelligence (AI) model was assessed for its utility in predicting vital signs of hemorrhage patients and optimizing the management of fluid resuscitation in mass casualties. With the use of a cardio-respiratory computational model to generate synthetic data of hemorrhage casualties, an application was created where a limited data stream (the initial 10 min of vital-sign monitoring) could be used to predict the outcomes of different fluid resuscitation allocations 60 min into the future. The predicted outcomes were then used to select the optimal resuscitation allocation for various simulated mass-casualty scenarios. This allowed the assessment of the potential benefits of using an allocation method based on personalized predictions of future vital signs *versus* a static population-based method that only uses currently available vital-sign information. The theoretical benefits of this approach included up to 46% additional casualties restored to healthy vital signs and a 119% increase in fluid-utilization efficiency. Although the study is not immune from limitations associated with synthetic data under specific assumptions, the work demonstrated the potential for incorporating neural network-based AI technologies in hemorrhage detection and treatment. The simulated injury and treatment scenarios used delineated possible benefits and opportunities available for using AI in pre-hospital trauma care. The greatest benefit of this technology lies in its ability to provide personalized interventions that optimize clinical outcomes under resource-limited conditions, such as in civilian or military mass-casualty events, involving moderate and severe hemorrhage.

## Introduction

Uncontrolled bleeding remains the major cause of preventable civilian and battlefield trauma deaths, with the greatest loss of life occurring in the pre-hospital environment (Eastridge et al., 2012; Kisat et al., 2013; Davis et al., 2014; Chang et al., 2016; Gurney and Spinella, 2018). The ability to identify and treat hemorrhage continues to be a top priority in combat casualty care, and attention is increasingly shifting toward conditions involving multiple casualties in austere and resource-constrained environments (Dolan et al., 2021; Lesperance et al., 2023). In these cases, artificial intelligence (AI) technologies represent a promising approach, providing decision support for triage, treatment, and resource prioritization at the lowest echelons of care (Raita et al., 2019; Fernandes et al., 2020; Liu et al., 2023; Peng et al., 2023).

The U.S. Department of Defense (DoD) has established practical population-based guidelines, procedures, and protocols to help combat medics identify and treat trauma-induced hemorrhage and provide fluid resuscitation in accordance with signs and symptoms, such as those provided by the Vampire Program (Voller et al., 2021) and the Tactical Combat Casualty Care guidelines (Deaton et al., 2021). These protocols encode robust and tried procedures that optimize outcomes when resources are readily available. Although they are based on population studies and do not provide patient-specific recommendations, these guidelines represent state-of-the-art field care, designed to support trained medics.

Machine-learning methods have been proposed to support the automation of casualty treatment, such as closed-loop fluid resuscitation (Kramer et al., 2008; Rinehart et al., 2013; Marques et al., 2017; Jin et al., 2018; Gholami et al., 2021; Alsalti et al., 2022; Avital et al., 2022). Although these treatments can potentially optimize fluid resuscitation for one casualty at a time, they do not address the simultaneous management of multiple casualties under resource-constrained conditions. Similarly, for the unstructured pre-hospital and the structured hospital environments, machine-learning methods have been developed to predict the need for life-saving interventions (Liu et al., 2014), including massive blood transfusion (Mina et al., 2013; Hodgman et al., 2018; Lammers et al., 2022), and to automatically analyze vital-sign data and stratify hemorrhage risk in trauma casualties (Stallings et al., 2023). While these methods flag the need for treatment, they do not necessarily provide a personalized resuscitation plan for each individual casualty.

Arguably, the main factor limiting the development of data-driven AI solutions for field care and triage of hemorrhage injuries is the lack of well-annotated and curated data to train these algorithms. In particular, deep neural network approaches used in the most powerful applications, such as large language models in medicine (Walker et al., 2023), require depth and breadth of quality data to become reliable. Here, we addressed this problem by using synthetic data derived from our previously developed cardio-respiratory (CR) mathematical model (Jin et al., 2023) to simulate multiple moderate to severe hemorrhage and fluid resuscitation scenarios, which we used to develop a recurrent neural network model capable of predicting treatment outcomes for each casualty 60 min into the future based on just 10 min of vital-sign data as input to the AI model. We then contrasted a resource-allocation method that used the AI-predicted outcomes with the DoD’s Vampire Program (Voller et al., 2021) to understand the prospective value and benefits of using such a method in mass-casualty scenarios and various resource-limited conditions. We hypothesized that we could use the neural network-based AI technologies in hemorrhage treatment to more efficiently allocate fluids to optimize clinical outcomes.

## Materials and methods

### Cardio-respiratory model

We used the CR model (Jin et al., 2023) to generate synthetic data that capture the time-dependent evolution of vital signs associated with hemorrhage and subsequent fluid resuscitation treatments. The CR model integrates cardiovascular and respiratory processes with their regulatory mechanisms to provide physiologically appropriate vital-sign time-course data that mimic the human response to hemorrhage and related treatments. The model consists of 74 ordinary differential and algebraic equations with 74 parameters. The inputs to the model include the rate of hemorrhage, rate of fluid resuscitation, minute ventilation, and fraction of inspired oxygen; the model outputs consist of arterial blood pressure [systolic (SBP), diastolic, and mean], heart rate (HR), partial pressure of end-tidal carbon dioxide, and oxygen saturation.

The CR model utilizes a lumped-parameter formulation based on first principles (conservation of mass) to represent fluid balances within vascular compartments and gas balances within the lungs and tissues, as well as a compartmental phenomenological formulation to represent the regulatory mechanisms and couplings between the cardiovascular and respiratory modules. Through this framework, the CR model enables the simulation of hemorrhage, fluid resuscitation, and respiratory perturbations, facilitating the generation of synthetic data that simulate injury and treatment scenarios of interest. It is important to note that a current limitation of the CR model includes the inability to account for specific types of resuscitation fluids, as the model solely considers the volume of fluid administered. For a more comprehensive overview of the CR model’s formulation and implementation, we direct the reader to Jin et al. (2023).

### Outline of the methodology to develop and assess the AI algorithm for personalized resource allocation of hemorrhage casualties

**FIGURE 1:**
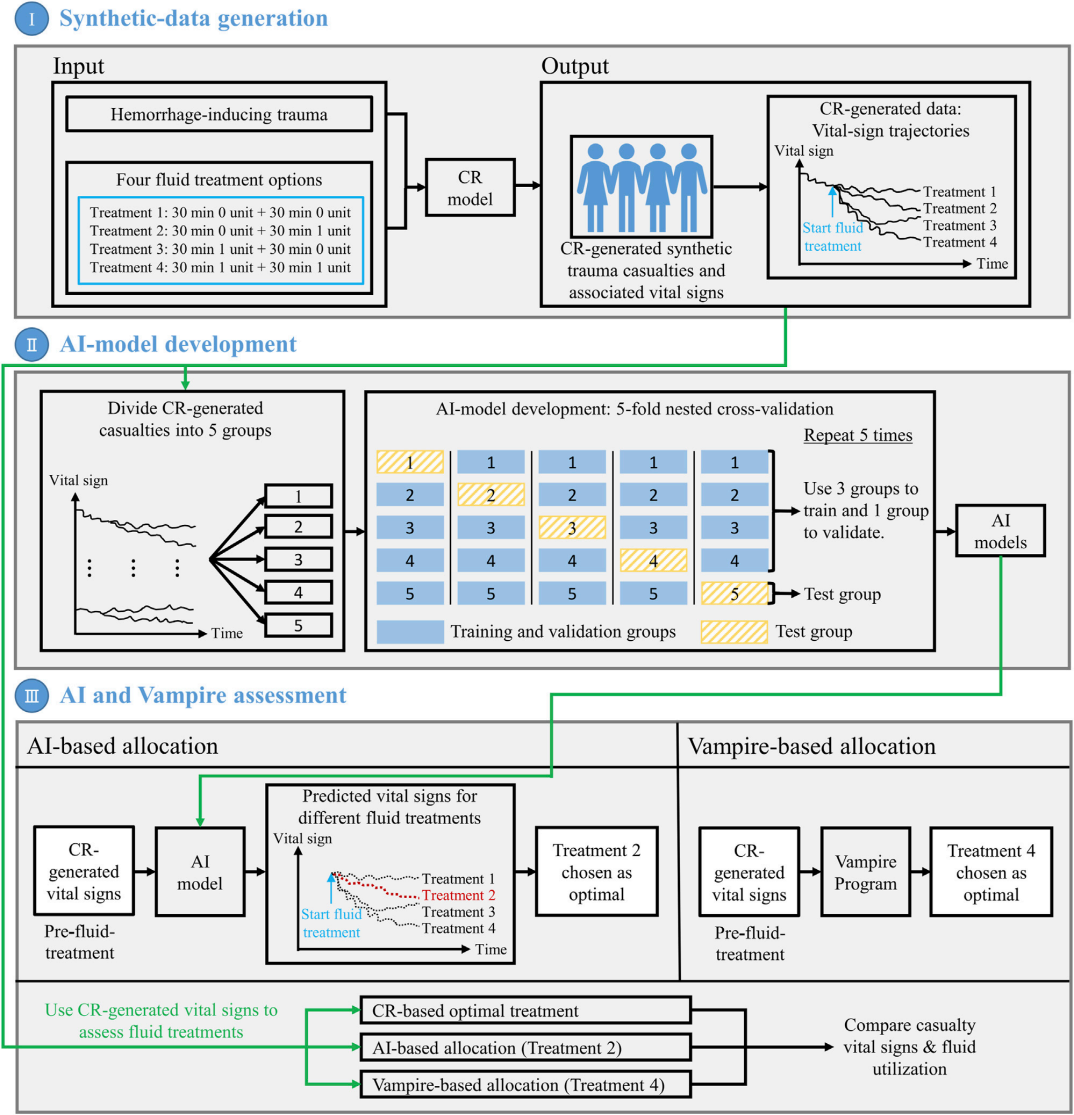
Outline of the methodology used to develop and assess the artificial intelligence (AI) algorithm for personalized resource allocation of hemorrhage casualties. I) Synthetic-data generation: Use the cardio-respiratory (CR) model to perform simulations and generate synthetic trauma casualties with associated vital-sign trajectories, for a given hemorrhage-inducing trauma condition and each of four fluid treatment options. II) AI-model development: Using the CR-generated synthetic data, perform a 5-fold nested cross-validation to develop AI models that use 10 min of pre-fluid-treatment vital signs to predict vital signs 60 min into the future after fluid treatment. III) AI and Vampire assessment: Use the CR-generated vital-sign data to compare the outcomes in terms of the number of restored casualties to a “safe” physiological state and the amount of fluid utilization for the optimal fluid treatments allocated by the AI model and the Vampire Program as well as the CR-based optimal fluid treatment.

We performed the three steps depicted in Figure 1 to develop and assess the AI algorithm for personalized resource allocation of hemorrhage casualties.

I) *Synthetic-data generation.* First, we performed simulations using the CR model to create synthetic vital-sign data and form a cohort of trauma casualties. The CR-model simulations generated vital-sign trajectories during an initial hemorrhage-inducing trauma, followed by four distinct fluid treatment options for each trauma casualty in the cohort. We considered this CR-generated synthetic data as the ground truth for subsequent analyses.
II) *AI-model development.* To develop the AI model, we first divided the entirety of the CR-generated cohort of trauma casualties into five equally sized groups (20% each). Then, we performed a 5-fold nested cross-validation procedure in which we iteratively trained and validated the AI model on data from four groups, and tested the model’s prediction performance on the data from the remaining fifth group. Through this process, we ensured that the AI model for each test group did not possess prior information regarding the vital signs of the casualties in that group. At the end of this iterative process, we obtained five AI models, one for each of the five groups, where we had trained the models such that, by using 10 min of pre-fluid-treatment vital-sign data, they could predict the vital sign outcomes 60 min into the future for each of the four fluid treatments.
III) *AI and Vampire assessment.* As a final step, we assessed the performances of the AI model and the Vampire Program (Voller et al., 2021) in their ability to optimize fluid allocation in trauma casualties in the test group. To determine the AI-based allocation, we provided the pre-fluid-treatment vital signs to the AI model, and used it to predict the vital-sign outcomes for each of the four fluid treatments. We used these predictions to choose the treatment (e.g., Treatment 2) that would lead to an optimal casualty outcome. To determine the Vampire-based allocation, we used its protocol to choose an optimal fluid treatment (e.g., Treatment 4) based on the pre-fluid-treatment vital signs. Finally, using the CR-generated vital-sign data, we compared the outcomes in terms of the number of restored casualties to a “safe” physiological state and the amount of fluid utilization for the optimal treatments chosen by the AI- and Vampire-based allocations as well as the “true” optimal treatment based on the CR-generated data. We repeated this procedure for each trauma casualty in each of the five test groups, using the corresponding AI model.

### Generation of hemorrhage and treatment scenarios

To generate synthetic vital-sign data for this study, we created variable sets of hemorrhage and treatment scenarios within defined injury, time, and fluid resuscitation limits. We created casualties corresponding to Class II and III hemorrhage (Schwartz and Holcomb, 2017), followed by a combination of tourniquet application and fluid transfusions commensurate with pre-hospital treatments documented in recent combat casualty care guidelines (Voller et al., 2021). The terms “bleeding” and “hemorrhage” with respect to the CR model correspond to a loss of fluid at a specific fixed rate for a specific length of time; to stop the bleeding, a “tourniquet” can be applied, corresponding to the bleeding rate set to zero in the CR model. This simplified hemorrhage case parallels extremity bleeding that can be controlled *via* the application of a tourniquet.

**FIGURE 2:**
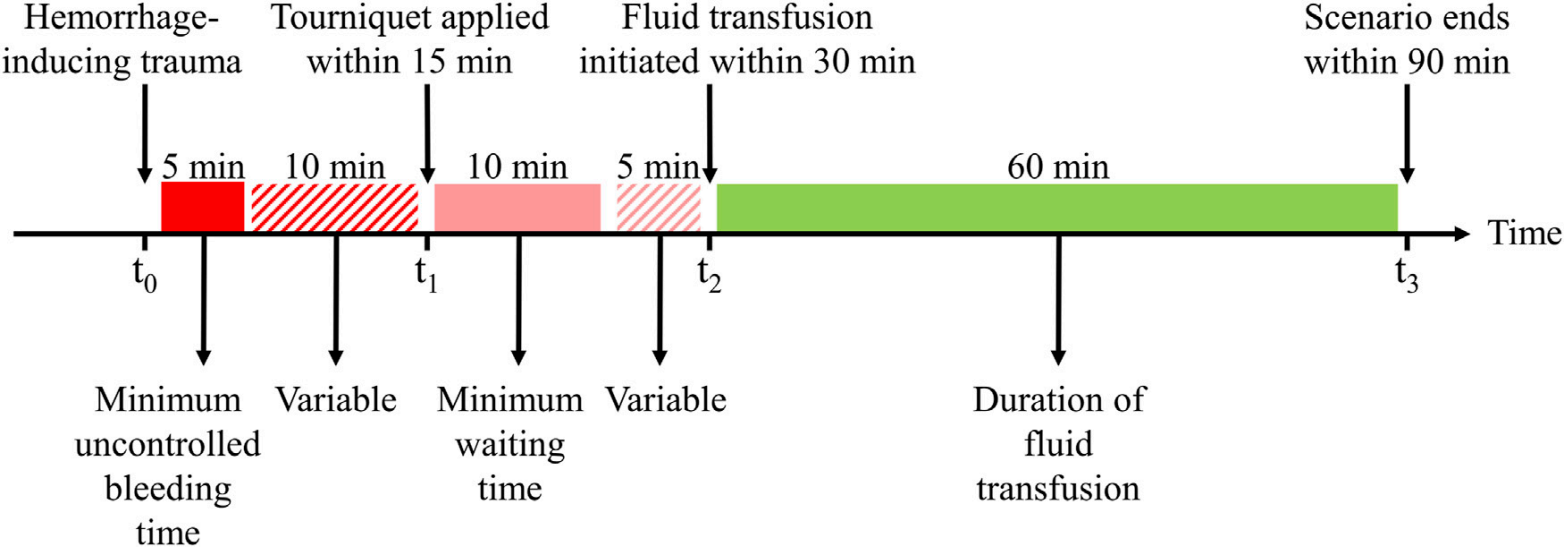
Events and time intervals used to create different scenarios representing an initial hemorrhage-inducing trauma, tourniquet application, and subsequent fluid resuscitation treatment. The injury at t_0_ is followed by a period of uncontrolled bleeding for a minimum of 5 min, after which a tourniquet is applied within a 10-min interval, i.e., from 5 to 15 min after the injury. The tourniquet application at t_1_ stops the bleeding, the fluid transfusion at t_2_ is initiated at a time interval 10–15 min after t_1_, and the transfusion continues for another 60 min until t_3_. Different scenarios sample different time intervals between t_0_ and t_1_ to apply a tourniquet and between t_1_ and t_2_ to start the transfusion, with blood transfusion starting between 15 and 30 min after the traumatic event. The maximum transfusion time is fixed at 60 min.

**FIGURE 3:**
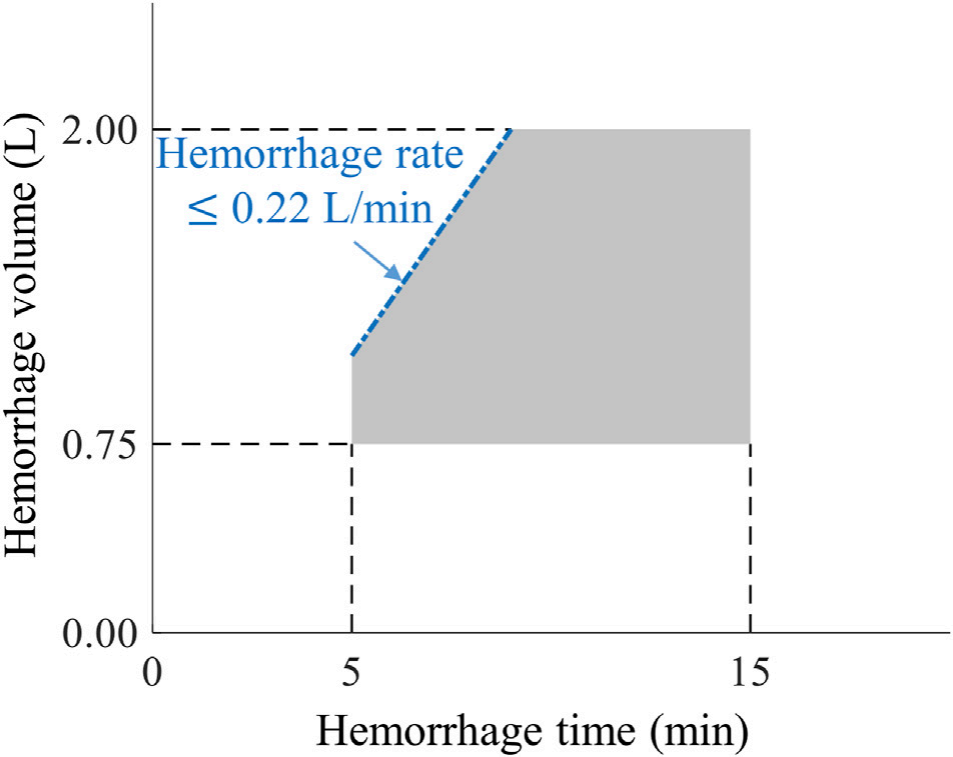
The range of bleeding times and blood-volume losses used to create different hemorrhage scenarios. The pentagon-shaded area defines the range of bleeding parameters used to create Class II and III hemorrhage cases (Schwartz and Holcomb, 2017) for this study, compatible with bleeding times of 5–15 min and blood-volume losses of 0.75–2.00 L. The blue dash-dotted edge of the pentagon represents the 0.22 L/min maximum rate of hemorrhage for these scenarios.

Figure 2 outlines the events and actions modeled in this work, i.e., the initial hemorrhage-inducing trauma occurs at t_0_, followed by the application of a tourniquet to stop compressible bleeding at t_1_, initiation of fluid resuscitation at t_2_, and completion of the scenario at t_3_. We then used a combination of fixed and variable time intervals between events to capture the range of temporal variability for treatment of combat casualties in a pre-hospital setting (Voller et al., 2021). To generate moderate (Class II) and severe (Class III) hemorrhage cases that induce noticeable changes in vital signs, we introduced a period of 5 min of uncontrolled bleeding followed by a variable period of 0–10 min before application of a tourniquet. This is compatible with the reported average pre-hospital tourniquet application time in recent conflicts (Kragh et al., 2009). In the CR model, tourniquet application corresponds to completely stopping further fluid loss. Hence, for each simulated scenario, the rate of fluid loss was based on the total volume of blood loss and the time up to tourniquet application. Figure 3 shows the range of possible blood-volume loss and bleeding time combinations that limit hemorrhage rates to the highest reported rate of 0.22 L/min (Herff et al., 2008; Soller et al., 2014; Kauvar et al., 2019).

Tourniquet application (t_1_) occurs within a maximum time of 15 min after the onset of hemorrhage, corresponding to no further fluid loss in the CR model. Subsequently, fluid transfusion is initiated at t_2_, which occurs within a variable time interval of 10–15 min after t_1_ (Figure 2). We chose this specific time interval based on two primary considerations: *1*) to ensure that the AI model had a minimum of 10 min of vital-sign data to learn the casualty’s physiological state to generate personalized predictions and *2*) to adhere to the military guideline that recommends the initiation of fluid resuscitation within 30 min of the hemorrhagic event (Shackelford et al., 2021).

**FIGURE 4:**
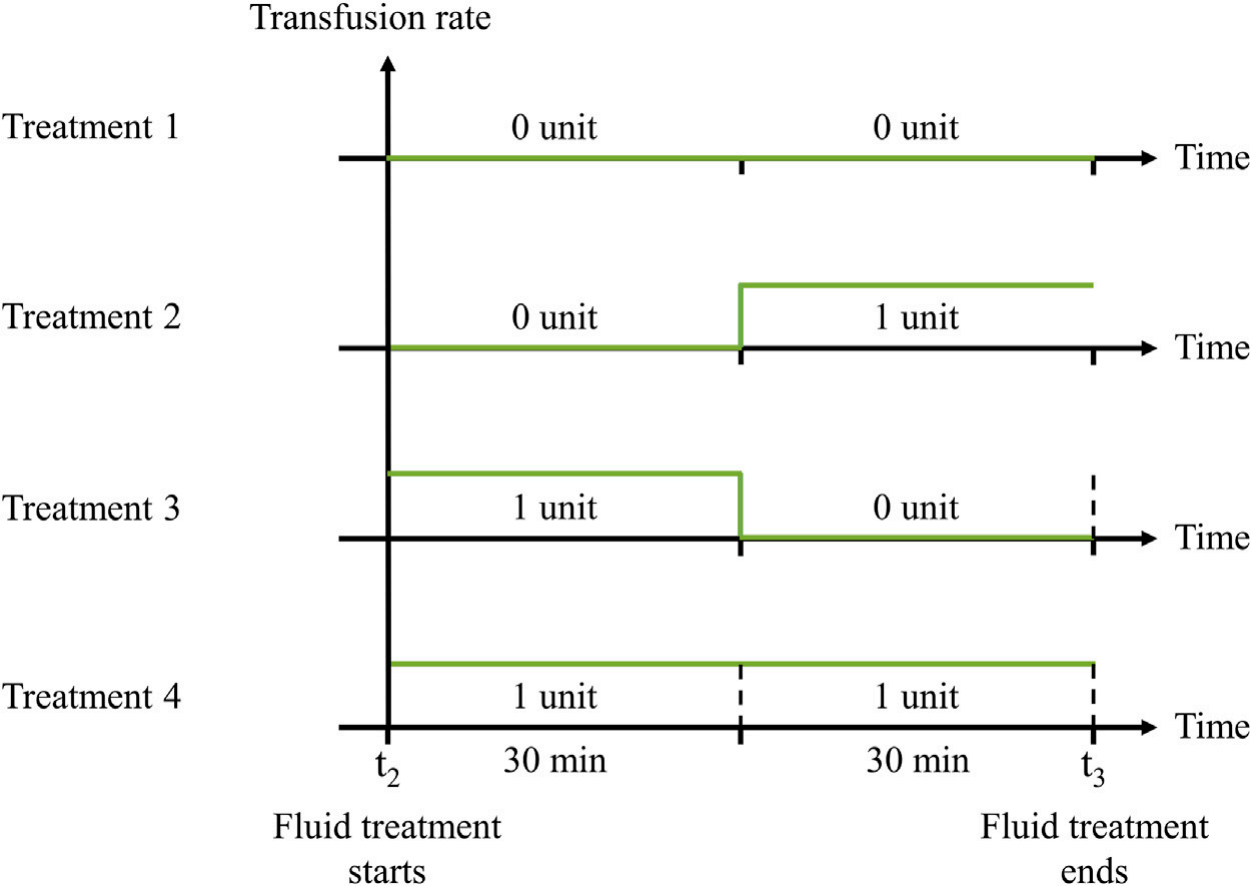
Transfusion rates of four fluid treatment options. 1) 30 min 0 unit + 30 min 0 unit; 2) 30 min 0 unit + 30 min 1 unit; 3) 30 min 1 unit + 30 min 0 unit; or 4) 30 min 1 unit + 30 min 1 unit. The fluid treatment starts at t_2_ and continues for 60 min until t_3_.

Taking into consideration that no more than 2 units of whole blood are generally administered within 60 min, with each unit containing an average volume of 0.55 L (Voller et al., 2021), we designed four distinct treatment options: 0 units for the entire 60 min; 0 units for the initial 30 min and 1 unit for the final 30 min; 1 unit for the initial 30 min and 0 units for the final 30 min; or 2 units sequentially administered in two 30-min intervals (Figure 4). For simplicity, we assumed a constant transfusion rate of 1.10 L/h. Finally, we assessed the status of the vital signs at t_3_, which occurs no more than 90 min after the onset of hemorrhage.

### Generation of synthetic data

**FIGURE 5:**
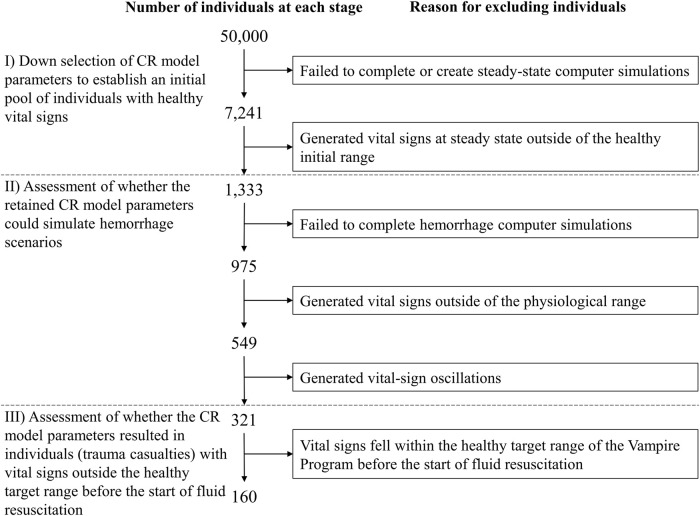
Procedure for selection of cardio-respiratory (CR) model parameter sets representing individuals used to generate vital-sign trajectories associated with the simulated hemorrhage and treatment scenarios. The selection procedure includes three different stages (I–III) in order to generate a broad range of individuals with vital signs in the healthy target range before hemorrhage and outside of this range after hemorrhage onset, to successfully simulate different degrees of moderate to severe hemorrhage.

We followed a three-stage procedure to select appropriate parameter sets for generating synthetic vital-sign time-course data with the CR model, corresponding to the scenarios depicted in Figure 2 and the range of hemorrhage parameters (volume, time, and rate) defined in Figure 3. We used this process to generate a broad range of parameter sets initially representing healthy individuals with varying initial vital-sign values that could be successfully simulated with different degrees of moderate to severe hemorrhage within the constraints discussed above. Figure 5 outlines the three stages described below.

I) *Down selection of CR model parameters to establish an initial pool of individuals with healthy vital signs.* We used the Latin hypercube sampling method (Helton and Davis, 2003) to generate 50,000 unique model parameter sets, each representing a unique individual, by randomly sampling the 74 CR model parameter values within ±70% of their nominal values. Then, we selected minimum and maximum HR values to define a “healthy” initial range, i.e., HR values higher than 60 beats/min (bradycardia) (Ou et al., 2016) and lower than 100 beats/min, as used in the Vampire Program (Voller et al., 2021). Similarly, we defined the healthy initial range for SBP as values lower than 140 mmHg (hypertension) (Gupta, 2004) and higher than 100 mmHg, as used in the Vampire Program. We only retained parameter sets with resulting vital signs in the healthy initial range.
II) *Assessment of whether the retained CR model parameters could simulate hemorrhage scenarios.* We tested whether the selected parameter sets, where each parameter set represents one individual, could simulate the five bleeding scenarios defined by the five vertices of the pentagon-shaded area in Figure 3, which describe the outer limits of blood-volume losses and bleeding times. We applied these five scenarios and excluded parameter sets that *1*) failed to complete hemorrhage simulations; *2*) resulted in vital signs outside of the physiological range (40 ≤ HR ≤ 200 beats/min and 40 ≤ SBP ≤ 260 mmHg), according to the ranges of vital-sign monitors (Mazoteras-Pardo et al., 2022; Peprah et al., 2023); or *3*) generated vital-sign oscillations.
III) *Assessment of whether the CR model parameters resulted in a final pool of N*_*F*_ *individuals* (*trauma casualties*) *with vital signs outside of the “healthy” target range before the start of fluid resuscitation* (*t*_*2*_)*.* Specifically, we aimed to eliminate casualties who fell within the healthy target range of the Vampire Program [HR ≤ 100 beats/min and SBP ≥ 100 mmHg (Voller et al., 2021)] at t_2_, because they would not require fluid resuscitation based on the Vampire Program. Finally, we applied all four treatment options (0 units, 1 unit for the initial 30 min, 1 unit for the final 30 min, and 2 units) to each of the final pool of N_F_ casualties resulting in 4N_F_ trajectories for the development of the AI model.

### Structure of the AI model

**FIGURE 6:**
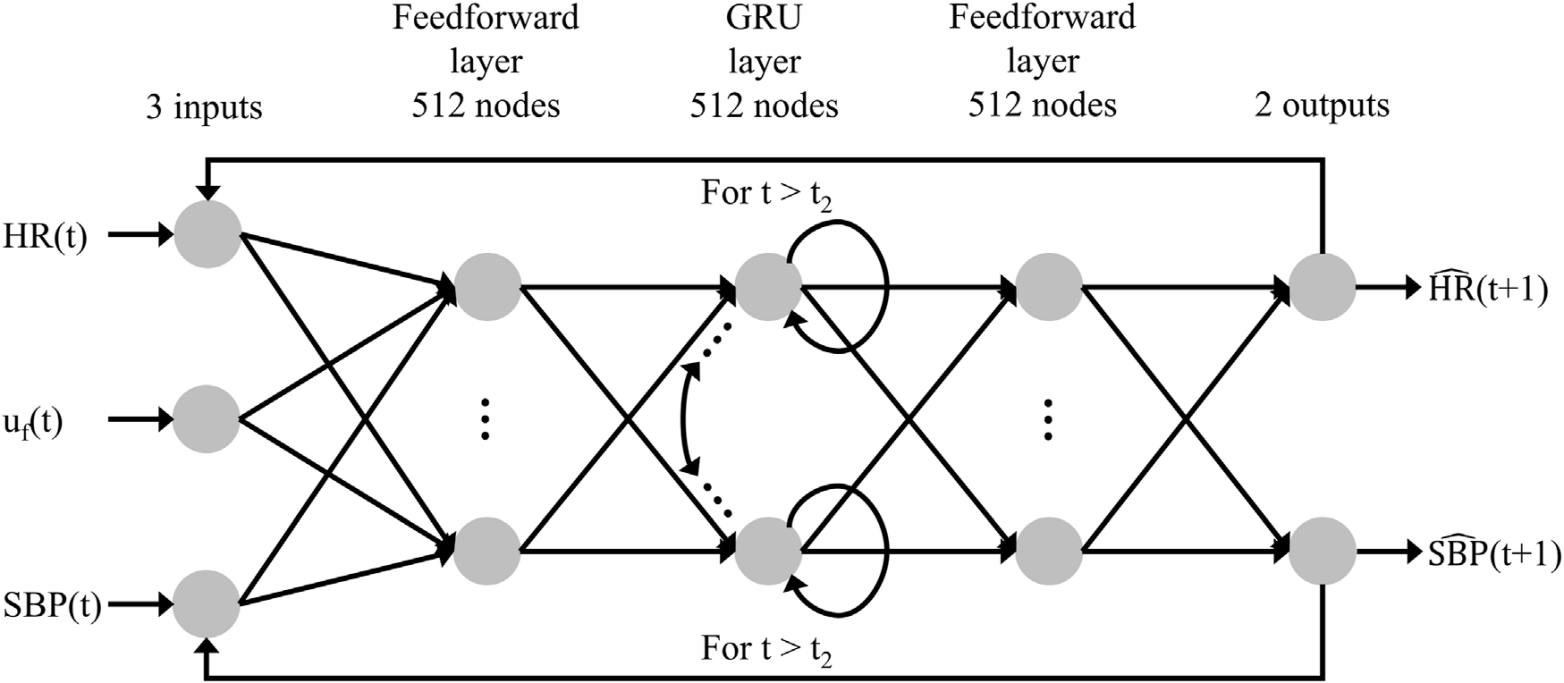
Structure of the recurrent neural network AI model. The model’s inputs are the fluid infusion rate [u_f_(t)], heart rate [HR(t)], and systolic blood pressure [SBP(t)] at time t, and the outputs consist of the predicted heart rate [ $\hat{\text{HR}}$ (t+1)] and the predicted systolic blood pressure [ $\hat{\text{SBP}}$ (t+1)] for the subsequent minute. The model architecture includes two feedforward layers and a gated recurrent unit (GRU) layer, each with 512 nodes.

To predict how trauma casualties would respond to fluid resuscitation, we developed a recurrent neural network model, namely, a gated recurrent unit (GRU) model (Cho et al., 2014), to predict the time-series evolution of HR and SBP. Figure 6 shows the overall architecture of the model, where unlike conventional feedforward networks that process each input independently, the GRU incorporates a “memory” mechanism that enables it to learn from a time series of inputs and update its hidden states accordingly. This functionality allows the model to capture the temporal dynamics inherent in vital signs.

At each time step t, the GRU receives three inputs: the fluid infusion rate [u_f_(t)], HR [HR(t)], and SBP [SBP(t)]. Consequently, it produces two outputs: the predicted HR [ $\hat{\text{HR}}$ (t+1)] and SBP [ $\hat{\text{SBP}}$ (t+1)] for the next time step (1 min in our case). The model architecture includes two feedforward layers and a GRU layer. To make personalized predictions, the GRU utilizes “measured” vital-sign data generated by the CR model from the preceding 10 min immediately before t_2_ (i.e., from t_2_–10 to t_2_), as defined in Figure 2, to update its hidden states. Starting at t_2_, the GRU continuously predicts $\hat{\text{HR}}$ (t+1) and $\hat{\text{SBP}}$ (t+1) for each subsequent minute by using u_f_(t) and the fed back values of $\hat{\text{HR}}$ (t) and $\hat{\text{SBP}}$ (t) until the scenario is completed at t_3_. Ultimately, we utilized the final predicted values $\hat{\text{HR}}$ and $\hat{\text{SBP}}$ at t_3_ to evaluate the treatment outcomes, thus providing insight into the efficacy of the applied intervention at t_2_.

### Development (training) of the AI model

The objective of the AI model is to use 10 min of vital-sign data before treatment of each casualty to predict the outcome of the corresponding treatment option 60 min into the future. We used the CR-generated vital signs and the corresponding fluid treatments to develop the AI model. To develop the AI model, we divided the cohort of N_F_ simulated trauma casualties into five groups of N_F_/5 casualties each and performed a 5-fold nested cross-validation procedure (Parvandeh et al., 2020), which simultaneously optimized the weights and hyperparameters of the model and assessed its performance. During the cross-validation procedure, we iteratively utilized three groups as the training set, one group as the validation set, and one group as the test set. Here, we trained the model with the aim of minimizing the sum of the normalized prediction error ɛ of vital signs (HR and SBP) over the 60-min duration of fluid resuscitation, as defined by Eq. 1 below:

$$ɛ=\sum_{t=1}^{60}\left\{{\;\left[\left[\hat{\text{HR}}\left(t\right)-\text{HR}\left(t\right)\right]/150\right]}^{2}+{\;\left[\left[\hat{\text{SBP}}\left(t\right)-\text{SBP}\left(t\right)\right]/110\right]}^{2}\right\}/60$$ (1)

where t denotes a time index; HR(t) and SBP(t) denote “measured” vital signs generated by the CR model; $\hat{\text{HR}}$ (t) and $\hat{\text{SBP}}$ (t) represent the predicted HR and SBP at time t, respectively; and 150 and 110 represent normalization factors indicative of the ranges observed during the CR-model simulations. To quantitatively evaluate the AI model, we also computed the root mean square errors (RMSEs) between the AI-model predictions and the CR-generated data over 60 min of fluid transfusion for HR (δ_h_) and SBP (δ_s_) of the training, validation, and test sets, as defined by Eq. 2a and Eq. 2b below:

$$\delta_{h}=\sum_{t=1}^{60}{\;\left[\hat{\text{HR}}\left(t\right)-\text{HR}\left(t\right)\right]}^{2}/60$$ (2a)

$$\delta_{s}=\sum_{t=1}^{60}{\;\left[\hat{\text{SBP}}\left(t\right)-\text{SBP}\left(t\right)\right]}^{2}/60$$ (2b)

For details on the development of the AI model, we direct the reader to Supplementary Text S1.

### Fluid allocation

We used the AI model to optimize fluid allocation and evaluated its performance by comparing it with the Vampire Program, a DoD guideline used to guide fluid resuscitation based on HR, SBP, and the presence of amputation. However, because the CR model only predicts vital signs, our analysis focused solely on HR and SBP. For the sake of simplicity, we modified the Vampire Program into a two-step process for our study: *1*) prior to fluid resuscitation, if the vital signs of the casualty were not within the healthy target range [HR ≤ 100 beats/min and SBP ≥ 100 mmHg], we initiated transfusion with 1 unit of fluid for 30 min and *2*) after the initial 30 min, we administered an additional unit of fluid if the CR-model-simulated vital signs continued to fall outside of the healthy target range.

Similarly, the developed AI-based fluid allocation strategy also consisted of a two-step process: *1*) before initiating fluid resuscitation, we employed the AI model trained on 10 min of data to predict the outcome of the casualty at 60 min for each of the four treatment options and selected the one that used the least amount of fluids to restore the casualty’s vital signs to the healthy target range. Then, we used the CR-generated data to obtain the outcome of the selected transfusion for the initial 30 min and *2*) after the 30 min, we used the available 40 min (10 + 30 min) of CR-model-simulated vital signs to update the AI model and predict the outcome at 60 min for each of two treatment options (0 or 1 unit for the final 30 min). Similarly, we selected the treatment that used the least amount of fluids to restore the casualty. When allocating fluids for a casualty within one of the five groups of N_F_/5 casualties, we used the AI model trained on the other four groups to predict the casualty’s vital signs. As a result, the models employed in our study do not possess any prior information regarding the casualties they are treating, ensuring a fair and unbiased allocation process. Moreover, to achieve the maximum number of casualties restored to the healthy target range with the given available fluid units, an optimal allocation strategy should refrain from administering fluids to casualties who do not require them or who cannot be restored to the healthy target range even with 2 units. Instead, the method should prioritize administering fluids to casualties in need of 1 unit, followed by those in need of 2 units.

To perform a side-by-side comparison between the AI- and Vampire-based allocation methods, we conducted three different analyses. *Analysis 1* served as a simple demonstration of the advantages offered by the AI allocation method, while the subsequent two analyses provided deeper insights into the relative performance and effectiveness of the two allocation methods under diverse scenarios.

*Analysis 1*. We employed the two methods to allocate fluids to one casualty and compared the number of used fluid units to restore the casualty to the healthy vital-sign target range.

*Analysis 2*. We expanded our evaluation by allocating varying units of fluids to N_F_/5 casualties within each group, employing both allocation methods. We first compared the number of casualties restored to the healthy target range for each of the two allocation methods as well as the CR-based allocation method, which provided an upper bound of the maximum number of possible restored casualties. Regarding the CR-based allocation method, we simply used the CR-generated data to obtain the outcomes at 60 min for all four treatments and selected the one that used the least amount of fluids to restore the casualty’s vital signs to the healthy target range. Similar to the AI-based allocation method, this method also prioritized administering fluids to casualties in need of 1 unit, followed by those in need of 2 units. Next, we compared the excessive use of fluids in the AI- and Vampire-based allocation methods (the number of fluid units used more than required based on the CR model).

*Analysis 3*. We explored the performance of the two allocation methods in a scenario involving the allocation of different units of fluids to varying numbers of casualties. To achieve this, we divided the N_F_/5 casualties of each group into different group configurations, including two groups of N_F_/10 casualties, four groups of N_F_/20 casualties, and eight groups of N_F_/40 casualties. Subsequently, we utilized both the AI- and Vampire-based allocation methods to distribute fluids to each group. We specifically examined the fraction of casualties restored to vital signs within the healthy target range using the AI-based method compared to the Vampire-based method. Additionally, we computed the relative ratio R of fluid-utilization efficiencies between the two methods, as defined by Eq. 3 below:

$$R=\left(N_{A}/U_{A}\right)/\left(N_{V}/U_{V}\right)$$ (3)

where N_A_ and N_V_ denote the total number of casualties restored to the healthy target range by the AI- and Vampire-based allocations, respectively, and U_A_ and U_V_ represent the total number of units of fluid utilized by the two methods. Hence, R > 1.00 indicates a greater efficiency of the AI method over the Vampire Program allocation. To prevent an “undefined” ratio R, we only evaluated R when at least 1 unit of fluid was used (i.e., U_A_ and U_V_ ≠ 0 units).

### Non-compressible bleeding detection

In the analyses above, we assumed that the tourniquet applied at time t_1_ set the bleeding rate to zero (completely stopped all bleeding) and that there was no non-compressible bleeding present. Here, we aimed to demonstrate the capability of the AI model to detect cases where the casualties experienced non-compressible bleeding. As hemorrhage typically leads to an increase in HR and a decrease in SBP, casualties with non-compressible bleeding are more likely to exhibit higher HR and lower SBP values (Henry, 2018). As the AI model does not account for non-compressible bleeding, the measured vital signs may deviate from the predicted values if bleeding persists. Therefore, by assessing the disparity between the measured (as predicted by the CR model, in our case) and the AI-model-predicted vital signs, it becomes possible to identify whether a casualty is still experiencing non-compressible bleeding or not.

To verify this capability, aside from the previously generated 4N_F_ trajectories referred to as the controlled bleeding scenario, we employed the CR model to generate an additional set of simulations for the cohort of N_F_ casualties. We conducted the simulations using the same bleeding rate but varied the fractions of non-compressible bleeding to 10%, 20%, 30%, 40%, and 50% of the total bleeding rate. Subsequently, we applied all four treatment options to these simulated trajectories, resulting in a total of N_N_ completed trajectories of non-compressible bleeding.

To classify the controlled and non-compressible bleeding scenarios, we utilized a support vector machine (SVM) with a linear kernel (Burges, 1998). Given the discrepancy in the number of trajectories between the two scenarios (4N_F_ trajectories for controlled bleeding and N_N_ trajectories for non-compressible bleeding), we weighted the trajectories inversely proportional to their respective numbers for classification, ensuring that trajectories from both scenarios contributed equally to the classification analysis. After implementing the SVM algorithm on the two scenarios, we computed the classification accuracy of each scenario to assess the performance of the detection method.

## Results

### Distribution of vital signs among selected individuals

We down-selected 50,000 individuals to a pool of 160 individuals (trauma casualties) with vital signs outside of the healthy target range of the Vampire Program (HR ≤ 100 beats/min and SBP ≥ 100 mmHg) before the start of fluid resuscitation (t_2_), through three stages. Figure 5 outlines the down-selection process. In particular, Stage I) established an initial pool of 1,333 individuals with healthy vital signs; Stage II) retained 321 individuals who completed the hemorrhage scenarios; and Stage III) assigned one random bleeding scenario within the shaded region in Figure 3 to each of the remaining 321 individuals and excluded 160 individuals whose vital signs fell within the healthy target range of the Vampire Program at t_2_. Finally, we randomly deselected one individual to generate a final cohort of N_F_ = 160 trauma casualties who could be evenly divided into five groups for data generation.

**FIGURE 7:**
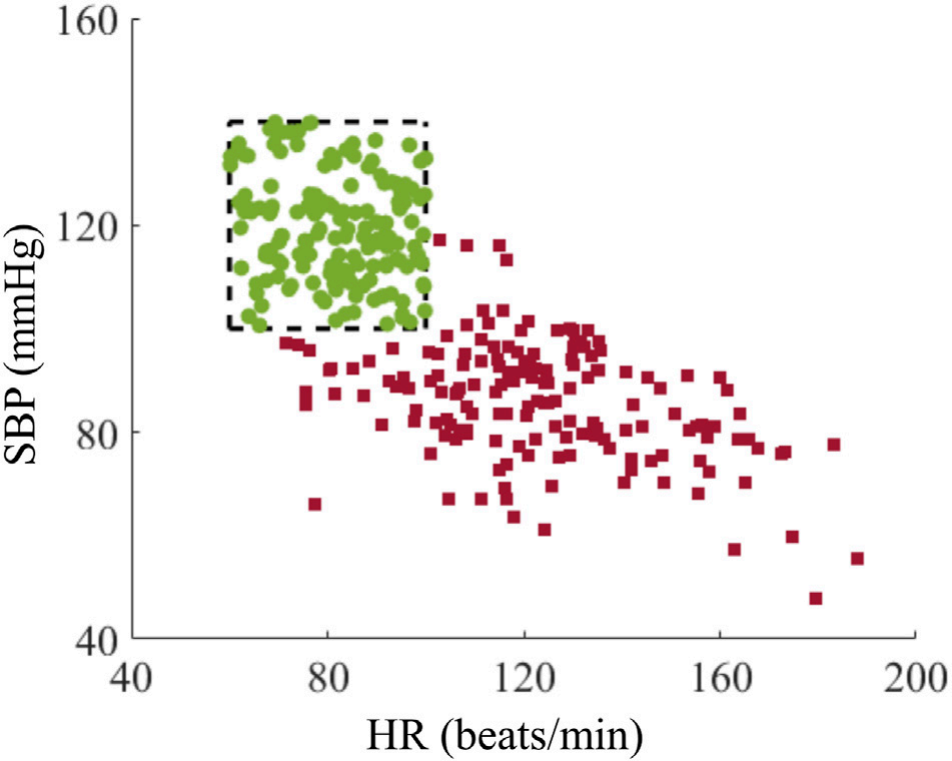
Distribution of heart rate (HR) and systolic blood pressure (SBP) for the cohort of 160 trauma casualties before (green circles) and after (red squares) hemorrhage. The range within the black dashed lines represents the healthy initial range.

To evaluate the range and variation of vital signs for the development of the AI model, we examined the distribution of their values before and after hemorrhage among the generated trauma casualties. Figure 7 shows the HR and SBP values for each of the 160 casualties at the beginning of the injury scenario (t_0_) in Figure 2, representing initial vital signs (green circles) in the healthy initial range and after hemorrhage at t_1_ (red squares). The initial vital signs were distributed across the entire healthy initial range, ensuring that the generated population captured a broad range of healthy baseline vital signs. In addition, the simulated hemorrhage scenarios led to an elevation in HR accompanied by a decrease in SBP, with HR values ranging between 70 and 200 beats/min and SBP values between 40 and 120 mmHg (Figure 7, red squares). The upper bound of HR and the lower bound of SBP spanned the range of their respective physiological limits, reflecting the ability of the injury hemorrhage scenarios to induce significant changes in vital signs. Thus, the generated data captured a high degree of individual variability, with a range of vital-sign changes that provided a diverse set of synthetic data for the development of the AI model.

### Training, validation, and test errors of the AI model

We divided the cohort of N_F_ = 160 trauma casualties into five groups of 32 (N_F_/5) casualties each and performed a 5-fold nested cross-validation. We first examined the three hidden layers of the recurrent neural network using 128, 256, and 512 nodes each and selected 512 nodes, as this consistently yielded the lowest average validation error ɛ between the AI-model predictions and the synthetic data over 60 min of fluid transfusion. For detailed results of the 128- and 256-node AI models, we direct the reader to Supplementary Figure S1.

Table 1 shows the average and standard deviation (SD) of the RMSEs between the AI-model predictions and the synthetic data for HR and SBP. As we used the 5-fold nested cross-validation method, we trained and validated 20 (5 × 4) AI models. The average training RMSEs of HR (δ_h_) and SBP (δ_s_) over the 20 models were 3.4 (SD = 0.9) beats/min for HR and 2.5 (SD = 0.7) mmHg for SBP. Likewise, the average validation δ_h_ and δ_s_ were 4.2 (SD = 1.0) beats/min for HR and 2.8 (SD = 0.5) mmHg for SBP. This correspondence between the training and validation errors indicated that the AI models were not over-fitted to the training data and generalized well to unseen validation data.

**TABLE 1:** Training, validation, and test root mean square error (RMSE) between the AI-model predictions and the synthetic data generated by the cardio-respiratory model over 60 min of fluid transfusion for heart rate (HR) and systolic blood pressure (SBP).

HR RMSE (δ_h_) (beats/min)			SBP RMSE (δ_s_) (mmHg)		
Training (N = 20)	Validation (N = 20)	Test (N = 5)	Training (N = 20)	Validation (N = 20)	Test (N = 5)
3.4 (0.9)	4.2 (1.0)	4.3 (0.7)	2.5 (0.7)	2.8 (0.5)	2.9 (0.5)

Data are presented as mean (standard deviation). N represents the number of AI models.

Finally, the average test δ_h_ and δ_s_ over the five groups were 4.3 (SD = 0.7) beats/min for HR and 2.9 (SD = 0.5) mmHg for SBP. Although these errors were larger than the validation error, the absolute errors were comparable to the level of vital-sign monitor instrumental accuracy, indicating that the AI model captured changes in HR and SBP associated with fluid resuscitation treatment of a broad range of hemorrhage scenarios in a population of diverse casualties.

### Performance comparison of fluid allocation methods

We conducted three analyses to evaluate the effectiveness of fluid allocations based on the AI predictions and the Vampire Program. Given that the CR model provides the ground truth for changes in vital signs upon hemorrhage as well as treatment, we compared both allocation methods to the CR model and assessed the relative performance of each method.

**FIGURE 8:**
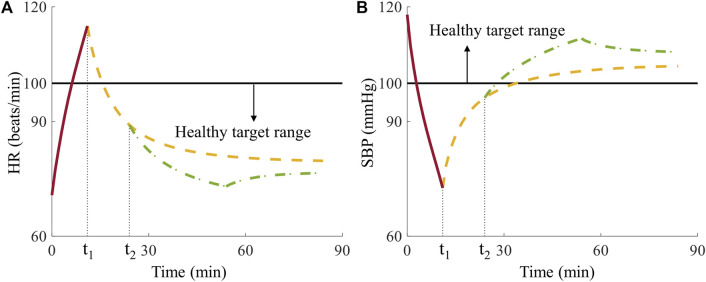
Comparison of Vampire- and AI-based allocation methods for the single casualty in *Analysis 1*. **(A)** Heart rate (HR) and **(B)** systolic blood pressure (SBP) over time, where t_1_ denotes the time for tourniquet application and t_2_ represents the time for initiation of fluid resuscitation, where the horizontal black solid lines represent the boundaries of the healthy target range. The red solid lines represent vital signs during the hemorrhage phase, the yellow dashed lines denote vital signs with no fluid transfusion, and the green dash-dotted lines represent vital signs after receiving 1 unit of fluids at t_2_ infused for 30 min during the treatment phase.

In *Analysis 1*, we examined fluid allocation methods using one casualty, which like all simulated casualties had vital signs at time t_2_ outside of the healthy target range of the Vampire Program (Figure 8). We selected this case to highlight one possible advantage of the AI-based method, where the best fluid allocation to restore the casualty to the healthy target range according to the CR model was by giving the casualty 0 units of fluid because tourniquet application alone at time t_1_ was sufficient. Figure 8 shows that at t_2_ the casualty’s HR fell inside the healthy target range (Figure 8A) while the SBP did not (Figure 8B). Consequently, the Vampire Program guideline initially recommended transfusing 1 unit of fluid for the initial 30-min period. Following this period, the vital signs returned to the healthy target range (Figure 8) and the guideline recommended discontinuing the resuscitation. In contrast, the allocation choice using the AI model was based on initially predicting the outcomes of all four treatment options for the casualty. Thus, the AI model correctly predicted that all four treatment options would result in outcomes within the healthy target range and, hence, no fluid was required for this casualty.

The ability of the AI-based allocation method to choose the optimal allocation strategy at the outset and ignore fixed vital-sign guidelines for fluid resuscitation allowed us to correctly transfuse 0 units of fluid to the casualty and return it to a healthy vital-sign state, while saving fluids. The predicted upfront knowledge of treatment outcomes provided the AI-based allocation a clear advantage in this case. However, the AI-based allocation method does not always outperform the Vampire Program because the model-predicted vital signs have small errors when compared to the synthetic data generated by the CR model.

In *Analysis 2*, we used a fixed number of casualties (N_F_/5 = 32) and introduced a varying number of available fluid units (0–42) for resuscitation. We compared *1*) the total number of casualties restored to the healthy target range by the two allocation methods compared to the CR model and *2*) the excessive recommendation and use of fluids generated by the allocation methods. To make a statistical comparison, we used the average results derived from the five groups, each made up of 32 casualties.

**FIGURE 9:**
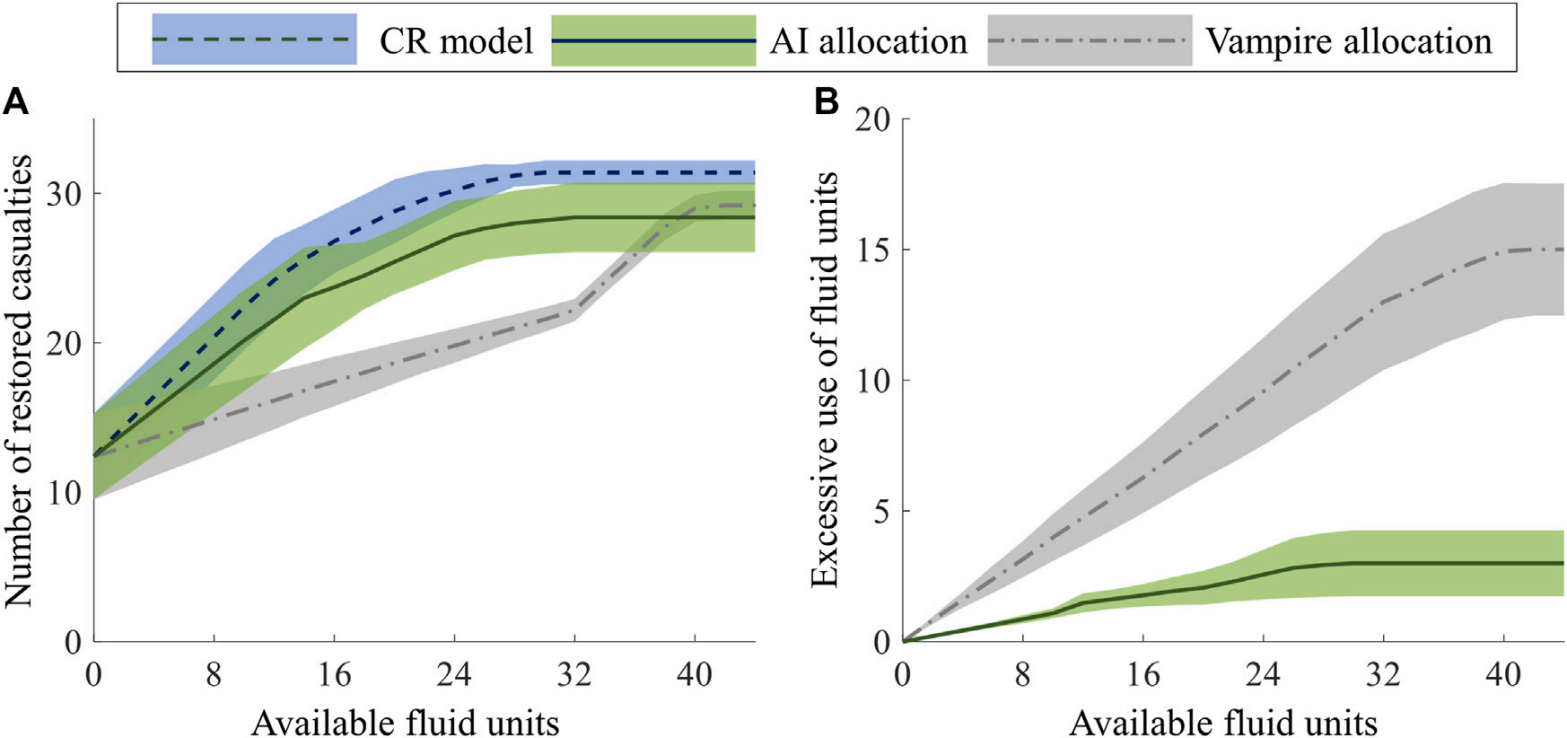
Comparison of fluid allocations based on the cardio-respiratory (CR) model, AI predictions, and the Vampire Program for different numbers of available fluid units. **(A)** Number of casualties restored to the healthy target range. **(B)** Excessive use of fluid units (number of fluid units used more than required based on the gold-standard CR results). The shaded areas represent two standard errors of the mean.

Figure 9A shows the number of restored casualties for the allocation methods based on the CR model (dashed line, blue shaded area), AI predictions (solid line, green shaded area), and the Vampire Program (dash-dotted line, gray shaded area). The lines and shaded areas represent the mean and two standard errors (SEs) of the mean. On average across the five groups, 12.4 casualties (2 SE = 2.9) were restored to the healthy target range without any fluid administration. As we increased the number of available fluid units for resuscitation, the average number of restored casualties rose. For the CR model (our gold standard), the average number increased at a rate of 1.0 casualty/unit until we administered 10 units, resulting in 22.4 (2 SE = 2.9) casualties restored to the healthy target range. Subsequently, the rate decreased to 0.7 casualties/unit until 16 units, leading to an average of 26.8 (2 SE = 2.1) restored casualties. Further increments in fluid units resulted in a decline in the rate to 0.5 casualties/unit until 20 units, with an average of 28.8 (2 SE = 1.9) restored casualties. Beyond 20 units, the rate continued to decrease, eventually stabilizing at 31.4 (2 SE = 0.8) restored casualties with 30 units. In contrast, the AI-based allocation restored fewer casualties compared to the CR baseline for any given number of fluid units. This was attributed to errors in the AI-model predictions and the resulting sub-optimal fluid allocation compared to the CR model. The average number of casualties restored increased at a rate of 0.8 casualties/unit until 14 units, resulting in 23.0 (2 SE = 3.4) restored casualties. Then, the rate decreased to 0.4 casualties/unit until 24 units, with an average of 27.2 (2 SE = 2.3) restored casualties. At the saturation point of 32 units, the average number of restored casualties stabilized at 28.4 (2 SE = 2.3). Comparatively, the Vampire-based allocation restored fewer casualties than the AI-based allocation. The difference was consistently larger in resource-limited conditions where the number of units was below 32. The average number of restored casualties increased only at a rate of 0.3 casualties/unit until 32 units, resulting in 22.2 (2 SE = 0.7) casualties in the healthy target range. Subsequently, the rate sharply increased to 0.9 casualties/unit until 40 units, yielding an average of 29.0 (2 SE = 0.9) restored casualties. Beyond this point, the rate became negligible, and the average number of restored casualties stabilized at 29.2 (2 SE = 1.0) up to the maximum of 42 units used for resuscitation.

Figure 9B shows the excessive use of fluids for the two allocation methods compared to the true minimum number based on the CR model. The excessive use of fluid units increased as the number of available fluid units increased, i.e., the inefficiency increased with increasing availability of fluids. For the AI-based allocation, the average excessive use of fluid units increased roughly at a rate of 0.1 per available fluid units. It peaked at 3.0 (2 SE = 1.3) units with 30 units of available fluid and saturated at this level. Comparatively, the Vampire-based allocation exhibited a much larger excessive use of fluid units, which increased at a rate of 0.4 per available fluid units until 32 units, resulting in an average of 13.0 (2 SE = 2.6) units of excessive fluid. Upon reaching 42 units, the excessive use reached a plateau, stabilizing at an average of 15.0 (2 SE = 2.5) units.

In *Analysis 3*, we examined variations both in the number of available fluid units (0–42) and the number of casualties (4, 8, 16, and 32) potentially requiring fluid resuscitation. Table 2 shows the fraction of casualties restored to the healthy target range using the AI-based allocation method compared to the Vampire-based allocation. The results consistently demonstrated that the AI-based method was more efficient (fraction >1.00) in resource-limited conditions across different numbers of casualties. In the case of 32 casualties, corresponding to the data shown in Figure 9A, the fraction of casualties restored to the healthy target range increased steadily until reaching 24 units of available fluid. At this point, the fraction peaked at 1.37 (SD = 0.09), indicating that the AI-based allocation method restored 37% more casualties than the Vampire-based allocation. However, beyond this point, the fraction of restored casualties started to decrease, and the two allocation methods became comparable when a larger number of fluid units were available, resulting in a fraction of ∼1.00 after 40 fluid units.

**TABLE 2:** Fraction of casualties restored to healthy vital signs based on the AI-based allocation method compared to the Vampire-based allocation method.

		Available fluid units												
		0	2	4	8	12	16	20	24	28	32	36	40	44
Number of casualties	32	1.00	1.07 (0.01)	1.13 (0.02)	1.25 (0.05)	1.33 (0.07)	1.36 (0.08)	1.36 (0.07)	1.37 (0.09)	1.33 (0.11)	1.28 (0.13)	1.10 (0.10)	0.98 (0.09)	0.97 (0.08)
	16	1.00	1.15 (0.05)	1.25 (0.09)	1.37 (0.16)	1.38 (0.19)	1.31 (0.26)	1.01 (0.14)	0.98 (0.11)	^a^	^a^	^a^	^a^	^a^
	8	1.00	1.27 (0.15)	1.37 (0.23)	1.30 (0.30)	0.98 (0.16)	^a^	^a^	^a^	^a^	^a^	^a^	^a^	^a^
	4	1.00	1.46 (0.60)	1.33 (0.59)	0.99 (0.23)	^a^	^a^	^a^	^a^	^a^	^a^	^a^	^a^	^a^

Fraction >1.00 indicates that the AI-based allocation method restored a larger number of casualties than the Vampire-based allocation method. Data are presented as mean (standard deviation) of the ratios of the number of casualties restored by AI-based allocation compared to those restored by the Vampire-based allocation for different numbers of available fluid units and casualties.

^a^ Indicates that the values are equal to the value on their left.

To gauge fluid-utilization efficiency, we compared the relative efficiency R in Eq. 3 (i.e., the number of casualties restored per utilized fluid unit) between the two methods. R values above 1.00 indicate that, on average, the AI-based method was more efficient than the Vampire-based method. Table 3 shows this fluid-utilization efficiency metric for variable numbers of available fluid units and hemorrhage cases potentially requiring fluid resuscitation. We observed that R ranged from 1.07 (SD = 0.01) to 2.19 (SD = 1.06), demonstrating a consistently improved fluid-utilization efficiency of the AI-based allocation method over the Vampire-based allocation. Similar to as in Table 2, the relative efficiency exhibited an increasing trend followed by a subsequent decrease, with the highest value achieved when the number of fluid units equaled the number of casualties.

**TABLE 3:** Relative fluid-utilization efficiency R of the AI-based allocation method compared to the Vampire-based allocation, where we computed the fluid-utilization efficiency as the number of casualties restored per utilized fluid units.

		Available fluid units												
		0	2	4	8	12	16	20	24	28	32	36	40	44
Number of casualties	32	1.00	1.07 (0.01)	1.13 (0.02)	1.25 (0.05)	1.33 (0.07)	1.44 (0.14)	1.53 (0.18)	1.55 (0.20)	1.64 (0.30)	1.77 (0.36)	1.70 (0.33)	1.67 (0.32)	1.66 (0.33)
	16	1.00	1.15 (0.05)	1.25 (0.09)	1.52 (0.27)	1.66 (0.36)	1.87 (0.56)	1.76 (0.56)	1.75 (0.56)	^a^	^a^	^a^	^a^	^a^
	8	1.00	1.27 (0.15)	1.64 (0.40)	2.04 (0.89)	1.90 (0.92)	^a^	^a^	^a^	^a^	^a^	^a^	^a^	^a^
	4	1.00	1.75 (0.69)	2.19 (1.06)	1.93 (0.96)	^a^	^a^	^a^	^a^	^a^	^a^	^a^	^a^	^a^

Data are presented as mean (standard deviation) of the relative efficiency R for different numbers of available fluid units and casualties.

^a^ Indicates that the values are equal to the value on their left.

### Non-compressible bleeding detection

**FIGURE 10:**
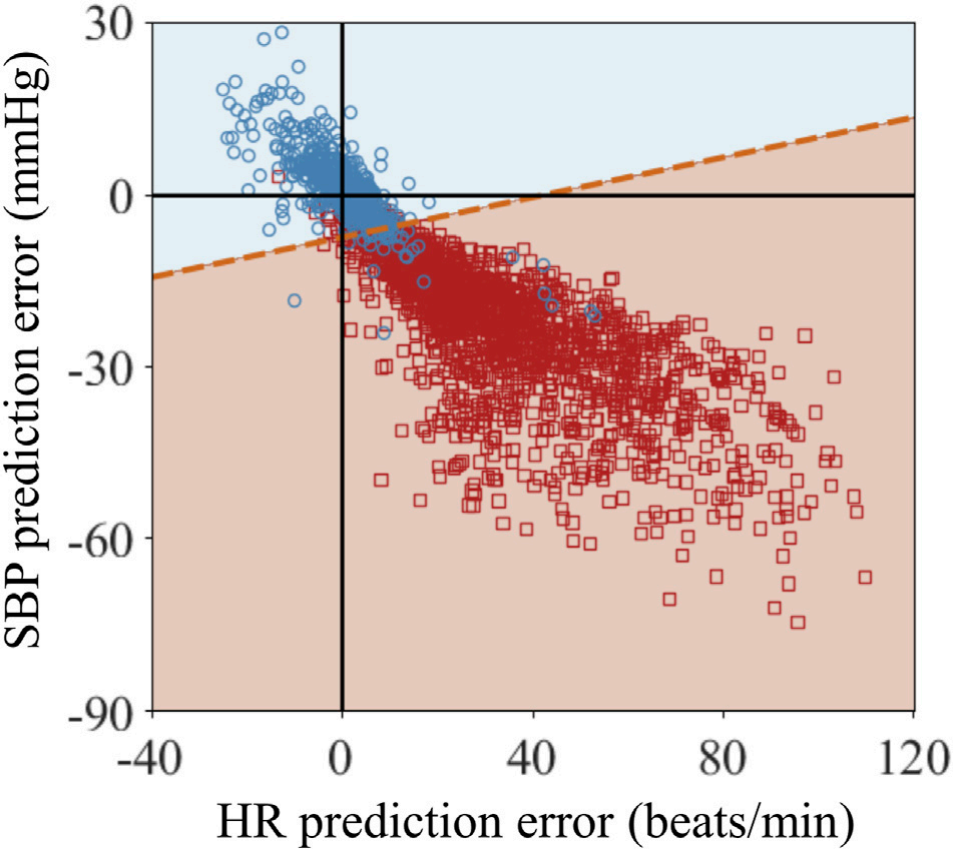
Classification results of the linear support vector machine algorithm for vital-sign trajectories at the end of fluid resuscitation at time t_3_ for the scenarios *1*) when tourniquet application at t_1_ controlled bleeding and *2*) when tourniquet application at t_1_ did not control all bleeding because there was additional non-compressible bleeding. The blue circles and red squares represent the prediction errors between the CR and AI models (CR minus AI) for heart rate (HR) and systolic blood pressure (SBP) at t_3_ for the two scenarios. The blue and red shaded areas represent the classified areas corresponding to controlled bleeding and non-compressible bleeding, respectively. The red dashed line between these two areas denotes the decision boundary that separates the two scenarios.

To verify if we could detect uncontrolled non-compressible bleeding, we generated 640 (4N_F_) controlled bleeding trajectories and N_N_ = 2,069 non-compressible bleeding trajectories. Figure 10 shows the classification results of the linear SVM in differentiating controlled *versus* non-compressible bleeding. The blue circles and red squares represent the prediction errors between the AI model and the CR model (CR minus AI) for HR and SBP at t_3_ for the controlled and non-compressible bleeding scenarios, respectively. As expected for the controlled bleeding scenario, the mean prediction errors for both vital signs were close to zero. Most of the prediction errors ranged from −22 to 26 beats/min for HR and from −24 to 27 mmHg for SBP (blue circles). In contrast, when non-compressible bleeding was present, it caused an increase in HR and a decrease in SBP, leading to corresponding changes in their prediction errors. The prediction errors ranged from −10 to 110 beats/min for HR and from −72 to 9 mmHg for SBP (red squares). This significant difference in the prediction errors between the two scenarios serves as a potential indicator for detecting uncontrolled non-compressible bleeding.

The blue and red shaded areas in Figure 10 represent the classified areas corresponding to the controlled and non-compressible bleeding regions, respectively, in the HR/SBP feature space. The red dashed line between the two areas denotes the decision boundary that separates the two scenarios. Because the decision boundary is neither horizontal nor vertical, both HR and SBP are essential for accurate classification. Quantitatively, the majority of the red circles and blue squares fell within their respective regions, indicating that the SVM accurately classified the bulk of the trajectories associated with the two scenarios. Table 4 shows the quantitative classification results of monitored trajectories in the two scenarios. We obtained an accuracy of 94% for the 640 controlled bleeding trajectories and an accuracy of 92% for the non-compressible bleeding trajectories, indicating the effectiveness of the AI model in detecting the presence of uncontrolled non-compressible bleeding.

**TABLE 4:** Classification results of the linear support vector machine algorithm for monitored trajectories at the end of fluid resuscitation (t_3_).

Scenario	Number of trajectories	Classified as		Classification accuracy (%)
		Controlled bleeding	Non-compressible bleeding	
Controlled bleeding	640	602	38	94
Non-compressible bleeding	2,049	165	1,904	92

Classification results are shown for monitored trajectories in the following two scenarios: *1*) when tourniquet application at t_1_ controlled any and all bleeding (Controlled bleeding) and *2*) when tourniquet application at t_1_ did not control all bleeding because there was additional non-compressible bleeding (Non-compressible bleeding).

## Discussion

The development, integration, and application of AI and machine-learning methods in medical care will be critical in future military conflicts to overcome anticipated challenges associated with high casualty rates, medical evacuation delays, and prolonged field care with limited resources. At all roles of medical care, the caregiver will need to efficiently match injuries with appropriate treatments under stressful conditions and with variable resource availability. Here, we examined the utility of a deep neural network model trained on time-series vital-sign data to predict trauma-induced hemorrhage outcomes and use this knowledge to optimize casualty treatment under resource-limited conditions.

As relevant clinical data of moderate to severe hemorrhage in the pre-hospital environment are scarce and insufficient for training AI models, the use of synthetic data is key in developing and understanding the behavior of such data-driven approaches. Hence, we used a computational cardio-respiratory response model to generate synthetic data representing the time evolution of vital signs after the onset of hemorrhage, followed by the application of a tourniquet and subsequent transfusion of resuscitation fluid. By varying the volume of blood loss and transfused fluid as well as the timing of events and model parameters, the CR model allowed us to generate a sufficiently large number of synthetic casualties that we used to train a recurrent neural network AI model. In addition, the computational CR model also served as the ground-truth physiological response for human casualties against which we compared the AI-model predictions. With these capabilities, we evaluated how AI solutions could impact assessment of hemorrhage injuries and associated treatment scenarios. In particular, we explored optimizing fluid allocations under resource-limited conditions and detecting uncontrolled non-compressible bleeding.

### Predictive performance of the AI model

To assess the predictive performance of the AI model, we compared its predictions with those of the ground-truth CR model results for HR and SBP. The AI model takes 10 min of initial vital-sign data after tourniquet application to make predictions of four different fluid-resuscitation treatments, ranging from no fluid up to 2 units of fluid in 60 min. The comparison of the predicted vital-sign trajectories against those of the CR data revealed low test errors (δ_h_ and δ_s_), with 4.3 beats/min (SD = 0.7) for HR and 4.1 mmHg (SD = 0.8) for SBP, indicating an overall accurate prediction of vital signs in response to different hemorrhage scenarios. The observed prediction errors were comparable to instrumental accuracy, and further attempts to reduce these errors could potentially lead to model overfitting and, hence, compromised model generalizability. While achieving zero errors would represent an ideal scenario where the AI-based allocation method perfectly matched the CR model-based allocation, it is important to strike a balance between prediction accuracy and generalizability.

### Performance comparison of the allocation methods

With the capability to prospectively evaluate treatment options based on limited initial vital-sign data and the AI model, we can select near-optimal fluid resuscitation treatment *before* starting the fluid infusion. This allowed us to construct a predictive allocation method that considered both the available resources and the number of casualties. To assess the performance of this AI-based allocation method, we conducted three analyses to compare it with the Vampire-based allocation method. Overall, the AI-based allocations outperformed the Vampire-based allocations in all three analyses, based on different performance metrics.

### Performance of non-compressible bleeding detection

We developed a linear SVM to distinguish between controlled bleeding and uncontrolled non-compressible bleeding, achieving high classification accuracies (>90%). These results highlight the effectiveness of the SVM in accurately distinguishing between these two bleeding scenarios, even as we considered a wide range of fractions (10%–50%) of uncontrolled non-compressible bleeding out of the total bleeding rate. As expected, as the fraction of non-compressible bleeding increased, it led to a more pronounced impact on HR elevation and SBP reduction, resulting in a relatively easier detection of non-compressible bleeding. Conversely, when the fraction of non-compressible bleeding was smaller, the corresponding changes in HR and SBP were less pronounced, making the detection task more challenging.

### Limitations and assumptions

Our work has several limitations arising from both practical needs and simplifying assumptions. Importantly, the lack of vital-sign, treatment, and clinical data from actual trauma casualties precluded their use to develop a deep recurrent neural network-based AI model. Instead, we used the CR model–proven to be relatively effective in capturing the dynamics of hemorrhage and associated treatments (Voller et al., 2021)–as the ground-truth gold standard to generate vital-sign data to train the AI model and compare allocation results. Although this approach does not exactly mimic real-world complications of moderate and severe hemorrhage, the design showcases the potential of using AI techniques to capture the complex dynamics of hemorrhage and treatment scenarios. The second limitation arises from a current constraint inherent in the CR model: because it does not simulate the effects of different fluid types and only uses a generic fluid volume, it cannot account for variations in fluid types. Although it is possible to enhance the CR model to incorporate different fluid types, further work would need to be conducted to enable a more comprehensive analysis of different availabilities of fluids and their optimal allocation. This limitation is also observed in other mathematical models (Bray et al., 2019), which may allow for the selection of different fluid types but result in the exact same change in vital signs. Another limitation of our study is the consideration of treatment outcomes for only 60 min of fluid resuscitation. We made this decision to simplify the optimization process. Nonetheless, it is worth noting that our AI approach, utilizing a recurrent network model, is capable of predicting vital signs at any given future time, although prediction accuracy would decrease with an increasing prediction horizon. Nevertheless, modifying our method to account for the assessment of treatment outcomes at different time durations is a feasible option, allowing for a more detailed analysis of the effectiveness of fluid allocation strategies throughout the resuscitation process. Finally, when applying the AI model for fluid allocation, we assumed that the CR bleeding rate became zero with the application of a tourniquet, no uncontrolled non-compressible bleeding or other complications were present, and no additional medical interventions were made. While these assumptions may not accurately represent real-world scenarios, it was necessary to isolate the effects of fluid resuscitation and simplify the evaluation of the allocation process. Future research could consider incorporating uncontrolled non-compressible bleeding as well as medical countermeasures to provide a more realistic representation of trauma scenarios.

## Conclusion

We assessed the utility of a deep recurrent neural network-based AI model to capture and predict vital signs associated with hemorrhage and fluid resuscitation and investigated how to use this model in creating optimal fluid allocations to handle mass-casualty scenarios, where resources are limited. Despite the limitations of a computational model of the cardio-respiratory response, the study design allowed us to develop insights into the caveats and utility of AI-directed medical decision-making. The importance of avoiding biased data is well known in the AI field, and the presence of bias and overfitting of vital signs in AI prediction models can only be overcome by careful selection of a balanced and varied set of casualties, hemorrhage rates, and fluid resuscitation options. Simply creating more data is not necessarily beneficial; the data must capture variable initial vital signs and represent a wide range of injury-treatment outcomes to benefit AI-model development.

Thus, our goal was not to use the CR model to generate data *per se*, but to create an application where a limited data stream (the initial 10 min of vital-sign monitoring) could be used to predict the outcome of different fluid resuscitation methods 60 min into the future. We then used knowledge of the possible outcomes to select optimal resuscitation strategies for mass-casualty scenarios under limited resource availability. Although the CR model represents a simplified hemodynamic cardiovascular response with numerous limitations, it does capture the correct coupled physiological behavior of HR and SBP variation during hemorrhage and fluid resuscitation. This allowed us to assess the potential benefits of using an allocation method that is based on personalized predictions of future outcomes *versus* a static population-based method that only uses currently available vital-sign information. The theoretical benefits of this approach include up to 46% additional casualties restored to healthy vital signs and up to a 119% efficiency increase in fluid utilization.

We further used the AI model to ascertain the error distribution of the predicted vital signs stemming from model imperfection due to training under the assumption that the bleeding rate was zero after tourniquet application. This is a valid assumption under a narrow set of conditions that exclude uncontrolled non-compressible bleeding or imperfections in the tourniquet’s ability to control any and all bleeding. If we compared this scenario to CR simulation results where we manipulated the bleeding rate and set varied fractions of uncontrolled non-compressible bleeding between 10% and 50%, we could investigate the same vital-sign error distribution resulting from the use of the AI model. As the two distributions are separable, we could, in theory, use the predicted results and compare them to the ground-truth data derived from the CR model, which represent the data that could be read from a vital-sign monitor, to flag discrepancies that indicate the presence of uncontrolled non-compressible bleeding not remedied by a tourniquet application.

While this study has limitations related to the use of synthetic data under specific assumptions, the work highlights the promise of integrating neural network-based AI technologies into the field of hemorrhage detection and treatment. The simulated injury and treatment scenarios revealed prospective advantages and potential applications of AI in pre-hospital trauma care. The primary strength of this technology stems from its capacity to provide personalized outcome optimization under resource-limited conditions, such as civilian or military mass-casualty scenarios.

## Data Availability

The raw data supporting the conclusion of this article will be made available by the authors, without undue reservation.
