# Supplementary material for: AI algorithm for personalized resource allocation and treatment of hemorrhage casualties
Source: Front Physiol. 2024 Jan 25;15:1327948. doi: 10.3389/fphys.2024.1327948 (PMC10851938; doi:10.3389/fphys.2024.1327948)
Supplement: Supplementary file 1 [file DataSheet1.docx]

**AI algorithm for personalized resource allocation and treatment of hemorrhage casualties**

**Xin Jin, Andrew Frock, Sridevi Nagaraja, Anders Wallqvist, and Jaques Reifman**

**Text S1. Procedure for the development (training) of the AI model**

We performed a 5-fold nested cross-validation approach to develop the artificial intelligence (AI) model through the following process consisting of four steps:

I) First, we divided the cohort of N_F_ simulated casualties into five groups of N_F_/5 casualties each. Given that the initial vital signs are associated with subsequent responses to fluid perturbations, we needed to ensure that we selected a balanced set of initial vital signs in each group to avoid biasing the model. Thus, we divided the healthy vital-sign target range, defined as heart rates (HRs) between 60–100 beats/min and systolic blood pressures (SBPs) between 100–140 mmHg, into four equally spaced regions and balanced each group to include an equal number of individuals in each quadrant.

II) Next, for the outer loop of the cross-validation, we treated one group as the outer test set and merged the other four groups to form the outer training set. We repeated this process iteratively for all five groups, ensuring that each group served as the test set once.

III) Then, within the outer training set, we established an inner loop. This involved training the AI model on three of the four groups within the outer training set and validating it on the remaining group. We repeated this training and validation process for all four possible combinations of groups. To optimize the weights of the AI model, we employed the Adam optimization algorithm (Jais et al., 2019) and aimed to minimize the sum of the normalized prediction errors ɛ of the vital signs (HR and SBP) over the 60-min duration of fluid resuscitation, as defined by Equation (1) below:

| $ɛ=\sum_{t=1}^{60} \left\{ \left[ [\hat{\mathrm{HR}}(t)-HR(t)]/150 \right]^{2}+\left[ [\hat{\mathrm{SBP}}(t)-SBP(t)]/110 \right]^{2} \right\}/60$ | $\text{(1)}$ |
| --- | --- |

where t denotes a time index; HR(t) and SBP(t) denote “measured” vital signs generated by the cardio-respiratory (CR) model; $\hat{\mathrm{HR}}$(t) and $\hat{\mathrm{SBP}}$(t) represent the AI-model-predicted HR and SBP at time t, respectively; and 150 and 110 represent normalization factors indicative of the ranges observed during the CR-model simulations. Notably, through experimentation, we observed that ɛ was predominantly influenced by the number of nodes in the gated recurrent unit (GRU) layer. Consequently, we used the same number of nodes across the three layers and tested different numbers of nodes (e.g., 128, 256, and 512), as plotted in Figure S1. For other hyperparameters, we set a 25% dropout rate for all three hidden layers, utilized a default learning rate of 0.001, and stopped the training process when the validation error did not improve over 2,000 epochs. We selected the optimal hyperparameters as those that yielded the lowest average validation error ɛ over the four inner models.

Ⅳ) Finally, to assess the prediction accuracy of the AI model, we created an ensemble model by averaging the predictions obtained from the four inner models with the best hyperparameters. We then tested this ensemble model on the corresponding outer test set. We repeated this process for all five groups, yielding five distinct test errors ɛ that allowed us to evaluate the model’s performance across different test sets. Moreover, to quantitatively evaluate the AI model’s performance in capturing the dynamics of HR and SBP during the fluid resuscitation process, we computed the root mean square error between the AI-model predictions and the synthetic data generated by the CR model for HR (δ_h_) and SBP (δ_s_) over 60 min of fluid transfusion, as defined by Equations (2a) and (2b) below:

| $\delta_{h}=\sum_{t=1}^{60} \left[ \hat{\mathrm{HR}}(t)-HR(t) \right]^{2}/60$ | $\text{(}\text{2a}\text{)}$ |
| --- | --- |
| $\delta_{s}=\sum_{t=1}^{60} \left[ \hat{\mathrm{SBP}}(t)-SBP(t) \right]^{2}/60$ | $\text{(}\text{2b}\text{)}$ |

**References**

Jais, I. K., Ismail, A. R., and Nisa, S. Q. (2019). Adam optimization algorithm for wide and deep neural network. *Data Sci. Eng.* 2**,** 41-46.

**Figure S1**. Average validation errors ɛ for different numbers (128, 256, and 512) of nodes used in the three layers of the artificial intelligence (AI) model. The five types of markers (blue triangle, green square, purple diamond, red star, and black circle) represent the average validation error ɛ for the five different groups of simulated casualties. To avoid over-parameterization, we did not consider models with a larger number of nodes as they would have led to an excessive number of model parameters. The AI model with 512 nodes in each layer consistently exhibited the lowest average ɛ, leading to its selection in the AI model.
